# Regulations of phage therapy across the world

**DOI:** 10.3389/fmicb.2023.1250848

**Published:** 2023-10-06

**Authors:** Qimao Yang, Shuai Le, Tongyu Zhu, Nannan Wu

**Affiliations:** ^1^CreatiPhage Biotechnology Co., Ltd., Shanghai, China; ^2^Department of Microbiology, Army Medical University, Chongqing, China; ^3^Shanghai Institute of Phage, Shanghai Public Health Clinical Center, Fudan University, Shanghai, China; ^4^Shanghai Medical College, Fudan University, Shanghai, China

**Keywords:** antimicrobial resistance, phage therapy regulation, clinical trial, investigational new drug, personalized phage therapy

## Abstract

Phage therapy, a century-long treatment targeting bacterial infection, was widely abandoned after the clinical availability of antibiotics in the mid-20th century. However, the crisis of antimicrobial resistance today led to its revival in many countries. While many articles dive into its clinical application now, little research is presenting phage therapy from a regulatory perspective. Here, we focus on the regulations of phage therapy by dividing sections into Eastern Europe where it was never abandoned and Western Europe, Australia, the United States, India, and China where it only re-attracted researchers’ attention in recent decades. New insights about its regulations in China are provided as little English literature has specifically discussed this previously. Ultimately, by introducing the regulations in phage therapy for human health across representative countries, we hope to provide ideas of how countries may borrow each other’s adapting legislation in phage therapy to best overcome the current regulatory hurdles.

## Introduction

The formal discovery of phage can be traced back to 1915 when Frederick Twort and Felix d’Herelle independently characterized its ability to cause “transmissible bacterial lyses” (Chanishvili, 2012). D’Herelle named it “bacteriophage” and started the first phage therapy in 1919 in Paris (Chanishvili, 2012). Following some successful cases, phage therapy was sensational for doctors and spread around the world (Chanishvili, 2012). However, it was widely abandoned since the commercial availability of penicillin in the mid-20th century, only remaining in Poland and the former Soviet Union (Lin et al., 2017; Fauconnier, 2019). Antibiotics, in contrast, had quickly taken over the market by virtue of their properties of standardized manufacture, administration, and their broad-spectrum antimicrobial ability.

Nevertheless, the extensive use of antibiotics brought increasing emergence and spread of antimicrobial resistance (AMR). What made the situation worse is the decreasing discovery rate of new antibiotics when the previous ones were rendered ineffective. The antibiotics crisis led to the renaissance of phage therapy in where it was once fully replaced by antibiotics. Increasing studies on the safety and efficacy of phage therapy in animals and humans were reported. Besides phage therapy that has been the standard of care in parts of the former Soviet Union for over 80 years, there are dozens of therapeutic phage pipelines entering phase 1–3 trials with investigational new drug (IND) approval in the West. However, in many countries experiencing the renaissance of phage therapy, there is still a lot of room for improvement regarding corresponding regulations and guidelines that may greatly facilitate the process. In this article, we will separately discuss the current and potential future regulations of phage therapy for human health in representative countries as an important reference for legislators, pharmaceutical companies, and researchers.

## Current progress in clinical phage therapy research

While there have been many investigator-initiated trials (IITs) of phage therapy since its discovery, more and more industry-sponsored trials (ISTs) that symbolize private investors’ commercial interest are emerging, representing investors’ growing confidence in this rapidly developing field.

### Investigator-initiated trials

From 1922 to 2022, phage therapy in more than 6,300 patients covered clinical departments of pneumology, urology, orthopedics, dermatology, otolaryngology, ophthalmology, gastroenterology, cardiology, and critical care medicine; involved treatments of infections of *Enterobacter*, *Acinetobacter*, *Pseudomonas*, *Staphylococcus*, *Enterococcus*, *Salmonella*, *Shigella*, *mycobacteria*, *Vibrio*, *Burkholderia*, *Serratia*, *Neisseria gonorrhoeae*, and other common clinical bacteria; and was implemented across the world in Europe, America, Asia, and Africa [summarized from Diallo and Dublanchet (2023)]. Phage therapy may bring promising clinical improvements and bacterial eradication results, while causing less or similar adverse events that are generally mild and resolved after the treatment than the control groups (Uyttebroek et al., 2022). In contrast to numerous case studies reported, randomized controlled clinical trials (RCTs) that are more in line with modern medical criteria are very limited, and many are primarily concerned with phage therapy’s safety. As shown in Table 1 with nine investigator-initiated RCTs published from 2005 to 2021. All these trials confirmed the safety of phage therapy relatively to the control groups. Also, several studies tested the efficacy of phage therapy compared to standard treatments (Wright et al., 2009; Sarker et al., 2016; Jault et al., 2019). However, the efficacy of phage therapy compared to the control groups demonstrated varying results, sometimes being superior but sometimes being inferior (Table 1). Though, these trials derived many suggestions for future studies, such as the possibility of raising phage dosage of administration to ensure effective working concentration and achieve better bacterial killing effect.

**Table 1:** The investigator-initiated RCTs of phage therapy.^*^

Name	Phase	Enrollment	Adm route	Target	Indication	Outcome	Developer	Ref.
WPP-201	1	42	Topical	*P. aerogenosa, S. aureus*, and *E. coli*	Chronic venous leg ulcers	Safety demonstrated	Southwest Regional Wound Care Center, United States	Rhoads et al. (2009)
Pyophage	2/3	113	IU	Multiple common bacteria	Urinary tract infection	Favorable safety profile, non-inferior to standard-of-care antibiotic treatment.	Balgrist University Hospital, Georgia	Leitner et al. (2021)
NS	1/2	24	Oral	*E. coli*	Chronic otitis	Safety demonstrated; clinical improvements; and bacteria reduction in the test group only.	Royal National Throat, Nose, and Ear Hospital, United Kingdom	Wright et al. (2009)
Microgen Coli Proteus and T4-like phage cocktail	NA	120	Oral	*E. coli*	Acute bacterial diarrhea	Safety demonstrated; no substantial intestinal phage replication; the test group showed no amelioration over control group.	Dhaka Hospital of the International, Bangladesh	Sarker et al. (2016)
PP1131	1/2	27	Topical	*P. aeruginosa*	Burn wound	Safety demonstrated; the control group reduced bacterial burden at faster pace	Burn centers in France and Belgium	Jault et al. (2019)
NS	NA	15	Oral	NS	Healthy	Safety demonstrated	Nestle Research Center, Switzerland	Bruttin and Brüssow (2005)
Microgen Coli Proteus	NA	15	Oral	*E. coli and Proteus*	Healthy	Safety demonstrated	Bangladesh	McCallin et al. (2013)
Microgen Coli Proteus	1	NS	Oral	*E. coli*	Healthy	Safety demonstrated	Bangladesh	Sarker et al. (2017)
PreforPro	NA	43	Oral	NS	Mild to moderate gastro-intestinal distress but no diagnosed	Safety demonstrated	Colorado State University, United States	Gindin et al. (2019)

^*^NS, Non-specified; NA, Not applicable; Adm. route, Route of administration; Ref., References; IU, Intraurethral/intravesical.

Account for any potential phage loss to achieve the optimal multiplicity of infection, allowing rapid phage replication to occur. In summary, these studies demonstrated the undoubtful potential of phage therapy but admitted that more systematic studies according to modern medical standards are needed.

### Industry-sponsored trials

Alongside the IITs that indicate the good performance of phage therapy, biotech and pharmaceutical companies are racing to develop new drugs based on formulated phage cocktails, phage endolysins, or even a phage collection. Table 2 highlights the current or planned ISTs of phage therapy reported to ClinicalTrials.gov since 2017. Several candidate pipelines have entered phase 3 clinical trials, and we expect to see commercial phage products available within 2–5 years. Worth mentioning, the United States Food and Drug Administration (FDA) revolutionarily approved INDs for Adaptive Phage Therapeutics’ personalized PhageBank therapy. Currently, the company has three pipelines of PhageBank therapy in phase 1–3 trials. Overall, most pipelines acquired their INDs in the United States, and other countries are relatively lagging in this stage.

**Table 2:** The ISTs of phage therapy since 2017 acquired from Clinicaltrials.gov.

Name	Phase	Adm route	Target	Indication	Starting year	Developer	Country	NCT. Id
Preforpro	3	Oral	NS	Vaginal infection	May 1, 2023 (est)	Deerland Enzymes	United Kingdom	NCT05590195
Pyobacteriophage	3	IH	Multiple common bacteria	Acute tonsillitis	October 2, 2020	Tashkent Pediatric Medical Institute	Uzbekistan	NCT04682964
LBP-EC01	2/3	IU & IV	*E. coli*	Urinary tract infection	July 13, 2022	Locus Bioscience	United States	NCT05488340
AP-PA02	2	IH	*P. aeruginosa*	Chronic pulmonary infection	January 10, 2023	Armata Pharmaceuticals, Inc.	United States	NCT05616221
PhageBank	2	NS	*S. aureus*	Diabetic foot osteomyelitis	November 24, 2021	Adaptive Phage Therapeutics, Inc.	United States	NCT05177107
PhageBank	2	IO	Multiple common bacteria	Chronic prosthetic join infection on hip/knee	March 27, 2023	Adaptive Phage Therapeutics, Inc.	United States	NCT05269134
PP1493 & PP1815	2	IA	*S. aureus*	Prosthetic joint infection	June 15, 2022	Pherecydes Pharma	France	NCT05369104
YPT-01	1/2	IH	*P. aeruginosa*	Cystic fibrosis infection	March 29, 2021	Yale University	United States	NCT04684641
BACTELIDE	1/2	Topical/IH	*S. aureus, P. aeruginosa*, or *K. pneumoniae*	Pressure ulcer infection	January, 2022 (est.)	Phagelux Inc.	United States	NCT04815798
PhageBank	1/2	IO & IV	Multiple common bacteria	Chronic prosthetic join infection on hip/knee	May, 2022 (est.)	Adaptive Phage Therapeutics, Inc.	United States	NCT05269121
BX005-A	1/2	Topical	*S. aureus*	Atopic dermatitis	May 2022 (est.)	BiomX	United States	NCT05240300
ShigActive	1/2	Oral	*Shigellosis*	Experimental Shigella challenge	February 23, 2023	Intralytix	United States	NCT05182749
EcoActive	1/2	Oral	*E. coli*	Crohn’s Diseases	May 1, 2019	Intralytix	United States	NCT03808103
VRELysin	1/2	Oral	*Enterococcus*	Intestinal infection	April 1, 2023 (est.)	Intralytix	United States	NCT05715619
NS	1/2	Topical	*S. aureus*	Diebetic foot ulcers infection	June 1, 2022 (est.)	Center Hospitalier Universitaire de Nîmes	France	NCT02664740
TP-102	1/2	Topical	*P. aeruginosa, S. aureus, A. baumanni*	Diabetic foot ulcer infection	March 22, 2021	Technophage	Israel	NCT04803708
WRAIR-PAM-CF1	1/2	IV	*P. aeruginosa*	Cystic fibrosis infection	October 3, 2022	National Institute of Allergy and Infectious Diseases	United States	NCT05453578
AP-PA02	1/2	IH	*P. aeruginosa*	Chronic lung infection	December 22, 2020	Armata Pharmaceuticals, Inc.	United States	NCT04596319
AP-SA02	1/2	IV	*S. aureus*	Bacteremia	April 26, 2022	Armata Pharmaceuticals, Inc.	United States	NCT05184764
BX004-A	1/2	IH	*P. aeruginosa*	Chronic pulmonary infection	June 21, 2022	BiomX, Inc.	United States	NCT05010577
PGX-0100	1	IH	*S. aureus, P. aeruginosa*, or *K. pneumoniae*	Burn infection	January 2022 (est.)	Phagelux Inc.	Australia	NCT04323475
BX002-A	1	Oral	NA	NA—Healthy individual	October 28, 2020	BiomX, Inc.	United States	NCT04737876
SNIPR001	1	Oral	NA	NA—Healthy individual	March 24, 2022	SNIPR Biome Aps.	United Kingdom	NCT05277350
PrePhage	1	Nasal	NS	Necrotizing Enterocolitis in Preterm infant	April 1, 2023 (est.)	Rigshospitalet Hospital	Denmark	NCT05272579

^*^Clinical trials that are canceled, discontinued, or have entered a new phase are not included. Information is up to date since the last check on June 25, 2023. NA, Not applicable; NS, Non-specified; Adm. route, Route of administration; NCT Id., Clinical Trial register ID; IV, Intravenous; IH, Inhalation; IO, Intraoperative; IU, Intraurethral/intravesical; and IA, Intra-articular injection.

## Regulations of phage therapy across the world

### Regulations in Poland, Georgia, and Russia

Although many countries only recently rediscovered their interest in phage therapy since the emergence of AMR crisis, it has always been widely used in Eastern Europe. In particular, phage therapy has been a part of the health care practice in Georgia, Poland, and Russia since its initial discovery (Międzybrodzki et al., 2018). Though, corresponding legislations as well as research and medical standards went through tremendous changes in recent decades to fit modern regulations more appropriately. In Poland, regulations in phage therapy became much more completed and systemized by falling under stricter administration and establishing ethical guidelines after Poland joined the EU in 2004 and established the Phage Therapy Unit (PTU) in 2005. Phage therapy in Poland was considered an experimental treatment based on several Polish legislations, particularly the Medical and Dental Professions Act of December 5, 1996 in addition to the Constitution of Poland and the ethical code of the Polish Association, and EU legislations on its member states (Żaczek et al., 2022). Phage therapy in Poland is also under Directive 2001/20/EC of the European Parliament and of the Council and Directive 2005/28/EC that regulate clinical trials and Good Clinical Practice (Hartmann and Hartmann-Vareilles, 2006). In Poland, the compassionate use (use of unapproved drugs to benefit patients) of phage therapy is together governed by the Helsinki Declaration and Guideline on Compassionate Use of Medicinal Products along with the Polish legislation (Żaczek et al., 2022). Worth noticing though, Directive 2001/20/EC was repealed in 2022 and replaced by European Medicines Agency (2023). While a transition period exists, it must be waited to see how this new legislation will impact clinical trials in phage therapy later. Based on the above regulations, phage therapy in Poland is now under systematic control, but it is only administered to patients in the PTU (Żaczek et al., 2022).

In contrast, phage therapy in Georgia and Russia is in an entirely different scenario. In Georgia and Russia, phage cocktails like “Intestiphage” and “Pyophage” can be directly purchased without a prescription (Abedon et al., 2011). However, their policies differ in many ways, especially regarding personalized phage therapy. In Georgia, pre-prepared phage and personalized phage medicines are considered pharmaceuticals. The pre-prepared phage medicines are under legislation for market authorization, while personalized phage medicines are allowed to be manufactured in pharmacies specially licensed by the Georgian Ministry of Healthcare through magistral preparation (Fauconnier, 2019). Phage products are similarly classified as pharmaceuticals in Russia, with manufacture and storage procedures listed in pharmacopeia GPM.1.8.1.0002.15 (Międzybrodzki et al., 2018; Pharmacopoeia.ru, n.d.). However, personalized phage therapy is forbidden, and only NPO Mikrogen is authorized by the State Register of the Ministry of Health of the Russian Federation to manufacture market medicinal phage cocktails (Vlassov et al., 2020). Furthermore, commercial phage products from Russia and Georgia are not recognized by the western medicine regulatory agencies, so exports of such products are difficult.

Hence, phage therapy is encountering developmental obstacles in these once-leading countries. Both countries, moreover, lack of double-blinded clinical trials, and potentially more regulations in this golden standard may improve their research progress in phage therapy (Abedon et al., 2011; Międzybrodzki et al., 2018).

### Regulations in the United Kingdom, France, and Belgium

In contrast to the long-term use of phage therapy in Eastern Europe, its application in Western Europe such as the United Kingdom, France, and Belgium is relatively scattered, but recent news demonstrated major advancements in its regulations in these countries. Since 2011, phage therapy was classified as a medicinal product by European Medicines Agency, but there were disputes about whether it should be classified as biological medicinal product according to Commission Directive 2001/83/EC or advanced therapy medicinal product based on Commission Directive 2003/63/EC (Naureen et al., 2020). However, because of its increasing importance, European Pharmacopoeia Commission sought to better specify phage therapy’s regulations by enacting a new general chapter named *Phage therapy active substances and medicinal products for human and veterinary use (5.31)* in 2021 and tasked this process to the newly established Bacteriophages Working Party (BACT WP). While the draft is being made, BACT WP is open to the public for advice until June 2023 (Council of Europe, 2023). This sets the precedence of modern mainstream medicine regulatory agency incorporating phage therapy in pharmacopoeia for human and veterinary use.

While there is no licensed phage therapy in the United Kingdom, the Medicine and Healthcare products Regulatory Agency regulates different aspects of phage therapy, classifying natural phage as biological medicine and overseeing the compassionate use of phage therapy (Jones et al., 2023). Under the regulation, domestically produced phage must follow GMP, and for clinical trials, only phages produced according to GMP may be used, while GMP is not required for imported phage for unlicensed use. Following more research, many proposals now advocate for more public funding for phage research and licensure coordinated by National Health Service (Jones et al., 2023). In response, the United Kingdom Parliament recently published an inquiry to draw evidence from different fields and experts to discuss the future of phage therapy and its regulations (UK Parliament, 2023). By the submission deadline, the inquiry received one oral transcript and 34 written evidence from various fields (Lin et al., 2023). Learning from the suggestions from the research institutes, healthcare providers, and capital interests within and beyond the United Kingdom, the parliament can hopefully gain a better understanding of the current situation of phage therapy and legislates accordingly to facilitate its applications.

Alongside following the EU guidelines as its member state, France also regulates phage therapy through the National Agency for the Safety of Medicines and Health Products (ANSM). Besides overseeing the compassionate use of phage therapy, the ANSM also formed temporary specialized scientific committees in 2016 and 2019 to investigate the potential of phage therapy in France (Procaccia, 2021). After research, the ANSM now treats phage therapy seriously and considers forming organized production and legal framework for it, pushing for the enactment of national guidance and approval platform for phage therapy in 2019 (Procaccia, 2021).

In contrast to the preliminary ideas in regulating phage therapy in the United Kingdom and France, Belgium features an established, innovative, and distinctive regulatory framework. Belgian Federal Agency for Medicines held the task to establish systematic regulations for phage therapy after Belgian Chamber of Representatives discussed the advantages and plights of phage therapy’s regulations in 2016 (Sacher, 2018). Following the discussion between different groups in Belgian, the government started to regulate phage therapy based on magistral preparation (Pirnay et al., 2018). In short, magistral preparation allows a pharmacist to produce medicinal products based on a physician’s prescription for each patient in pharmaceutical standards (Pirnay et al., 2018). Official pharmacopoeia such as European Pharmacopoeia and Belgian Pharmacopoeia guide the requirement for active ingredients in such a preparation (Fauconnier, 2018). The Minister of Public Health provides additional guidelines when information from those pharmacopoeia are insufficient, and a Belgian Approved Laboratory, which is an accredited quality control agent by the government, certifies whether non-authorized ingredients can be incorporated in the preparation (Fauconnier, 2018). Compared to many other countries in the EU, magistral preparation in Belgian allows tailored therapy to individual patients whereas the compassionate treatment can only be applied under emergency with exceptional nature.

### Regulations in Australia

Another country where research and health professionals are embracing phage therapy in recent years is Australia. Connected by Phage Australia, a national alliance aiming to systemize phage therapy, researchers and clinicians feature expansive network across the country based in centers like hospitals and research institutes. While the stakeholders are working toward professionalizing the therapy, it is not readily available to the public yet (Lin et al., 2019). However, currently three pathways, special access scheme (SAS), clinical trial notification (CTN), and clinical trial exemption (CTX), allow phage therapy to be administered after a referral process from family doctor or specialist to infectious disease specialist to Phage Australia (Lin et al., 2019; Bacteriophage.news, 2022). SAS serves like compassionate practice of using treatments that are not on Australian Register of Therapeutic Goods. CTN and CTX, on the other hand, are clinical trial pathways that differ in some ways. In CTN, the Human Research Ethics Committee and research sponsor are responsible for reviewing and approving the research protocol followed by clinical trial, and the Australian Therapeutic Good Administration (TGA) is notified. In contrast, CTX is the pathway in which TGA reviews the protocol and often involves novel treatments. What determines the procession of CTN or CTX is whether the Human Research Ethics Committee has the expertise to assess the safety of the treatment. If it has, then it follows CTX, or else it follows CTN. Furthermore, Phage Australia uniquely adopts the Standardized Treatment and Monitoring Protocol for Adults and Pediatric Patients (STAMP) protocol to evaluate the clinical process for phage therapy (Phage Australia, n.d.). This has immense implications as by studying and standardizing the process, different phages may be used, greatly facilitating personalized phage therapy. The STAMP process is paired up with SAS (Phage Australia, n.d.). With a national phage alliance, various pathways for phage therapy, and an innovative approach in focusing on the holistic process rather than a specific phage, Australia demonstrates its promising future in phage therapy.

### Regulations in the United States

In the United States, Office of Vaccines Research and Review in the Center for Biologics Evaluation and Research of FDA classified phage therapy as biological product and regulate it accordingly, and its manufacture must follow standards like GMP, preclinical research, and clinical trial (Furfaro et al., 2018). While there is no FDA-approved phage therapy yet, the United States has the most phage-related ISTs, some being at phase 3 clinical trial (Table 1). The first clinical trial assessing genetically modified phage was also approved in the United States (NCT05488340). Applications of INDs for phage therapy are similarly subjected to the review of FDA just as other drugs, and clinical trials may be started if FDA does not hold on the application after 30 days (Suh et al., 2022). Also, like the compassionate use of phage therapy elsewhere, FDA allows the extended access to the INDs of phage therapy under exceptional situations for patients when they cannot enroll in clinical trials (U.S. Food and Drug Administration, 2021). The Institutional Review Board and FDA must be notified about the extended use of phage therapy (Suh et al., 2022). Regarding personalized phage therapy, FDA made revolutionary progress by approving the IND of Adaptive Phage Therapeutics’ phage bank therapy, featuring the only phage bank therapy with IND approval in the world (Adaptive Phage Therapeutics, 2021).

### Regulations in India

While phage therapy in India just emerged in recent years, phage research can be traced far back when British scientists discovered unknown that killed cholera bacteria in 1896. The unknown is highly suspected to be phage as one of the first phage discoveries (Ranjith et al., 2019). In 1940, the government established the Central Drug Laboratory, Kasauli, to work on biological products including phage based on Drugs and Cosmetics Act (1940) (Ranjith et al., 2019). Phage therapy, however, was not much promoted until 2017 when Vitalis Phage Therapy was founded by Pranav Johri, who treated his multi-drug resistant infection using phage therapy in Eliava Phage Therapy Center in Georgia, and now cooperates with the center to provide phage therapy for others (Sacher, 2019). Today, because no clear regulations is made regarding phage therapy, it in India is offered as compassionate Phage Therapy (cPT) on the compassionate base and is regulated by the Declaration of Helsinki and coordinated by the Central Drugs Standard Control Organization that enables cPT in India as it allows the importation of unregulated drugs like phages for compassionate treatment (Ranjith et al., 2019; Johri, 2023). Realizing the potential of phage therapy, the Indian government is trying to promote phage therapy as the Indian Council of Medical Research (ICMR) has been gathering phage researchers and stakeholders to discuss relevant details, so more specific regulations and research centers can be expected in India (Johri, 2023).

### Regulations in China

It was as early as 1958 when the Shanghai Jiao Tong University School of Medicine successfully treated a patient with *P. aeruginosa* burn infection using phage therapy (Liang et al., 2023). However, the ethics approval system was not yet established. It was not until 2019 when Shanghai Institute of Phage conducted the first IIT of personalized phage therapy at Shanghai Public Health Clinical Center and Zhongshan Hospital Fudan University under the ethics approval framework (Bao et al., 2020).

Related studies have also been conducted in Shenzhen later (Tan et al., 2021). Following the progress in Shanghai and several other hospitals in Shenzhen, Xi’an also launched IITs of phage therapy recently. However, no company has acquired IND to conduct IST in China. According to Chinese regulations, commercial phage therapy applications can go through two pathways. Firstly, phage products with fixed ingredients should be regulated as innovative biological products (Lu et al., 2023). Their INDs need to be submitted to the Center for Drug Evaluation (CDE) under National Medical Product Administration (NMPA), which are then subjected to IST regulated by Chinese Good Clinical Practice and Measures for the Administration of Drug Registration (Lu et al., 2023). Secondly, personalized phage therapies need to go through IIT under Management Measures for Clinical Research (Huang, 2022). Based on successful results, researchers from a medical institution may apply for restrictive medical technology in Provincial Health Commission, and upon approval, the personalized phage therapy can be conducted at certain institutions (Huang, 2022; Yang, 2023). Although phage-related legislations are yet to be created, legislators are increasingly aware of this rapidly developing field. For instance, On January 31, 2023, the Development and Reform Commission of Shenzhen Municipality issued three documents consecutively to emphasize the support that will be placed in nine core technologies that are facing bottlenecks, one being phage therapy (Development and Reform Commission of Shenzhen Municipality, 2023).

### Future prospects

In summary of the countries discussed above, it can be seen that compassionate use is a major pathway that patients rely on to access phage therapy overall, especially in the countries that started to embrace the therapy in recent decades. The representative countries in which the compassionate use being a major access to phage therapy include the United Kingdom, France, Belgium, Australia, India, China, and the United States despite that the terms that these countries use in referring to compassionate use may be different. It can be argued that even Poland, in which phage therapy has been a long tradition, practices the compassionate use of phage therapy after it adopted more related regulations as it currently defines phage therapy as experimental treatment and regulates it like compassionate treatment in many ways. In addition, phage therapy can also be accessed if clinical trial is available in many of these countries. However, given the considerable demand and various types of research involving clinical trials done, phage therapy demonstrates its enormous potential that made these countries to come up with more strategies in recent years to extend it beyond the scope of compassionate use. Among the new policies, Belgium features the most significant and innovative regulation by establishing a thorough regulatory framework for phage therapy, the unique magistral preparation in Western Europe, enabling the public access to phage therapy. The other countries are also working toward public access. Belgium’s counterparts, the United Kingdom and France, are on the way to innovate their current regulations for phage therapy by posing inquiries to specialists and forming expert committees. More broadly, the European Pharmacopoeia Commission announced to add a new general chapter specifically regulating phage therapy, and this will be the party’s primary goal from 2023 to 2025 (Council of Europe, 2023). India is on the similar track as the ICMR is also gathering professional perspectives and the local company has partnered with the experienced Eliava Institute. Besides, Australia formed a national alliance to promote the STAMP protocol that are similar to magistral phage in some ways, like focusing on the process rather than a particular product. Over the sea, the United States unprecedentedly granted INDs for personalized phage bank treatment to Adaptive Phage Therapeutics. In 2021, FDA held a series of conferences among phage experts to discuss the prospects and regulations of phage therapy (U.S. Food and Drug Administration, 2021). Recognizing the importance of phage therapy through ongoing compassionate treatments, the conference encouraged more well-controlled clinical trials to support the official licensure of phage therapy.

In this process of forming novel regulations for phage therapy, China seems to fall behind as no major turning point has occurred yet despite the government’s emphasis on supporting phage therapy at some regional levels. To further promote phage therapy, the governments and researchers should borrow regulatory experiences from other treatments. In general, the concerns related to the biological characteristics of phage can be addressed by learning from the regulations for treatments with similar characteristics, such as viral vector vaccines, oncolytic viruses, and neutralizing antibodies. Also, regulations in fixed-ingredient phage cocktails can be referred to other treatments that are similarly subjected to IST. For instance, cellular immunotherapy like Neoantigen-Targeting T Cell suspension for intravenous infusion uses a similar procedure as a phage cocktail to first get IND from CDE and conduct IST upon NMPA approval. Moreover, many personalized therapies including cellular therapy and fecal microbiota transplantation approved by the Provincial Health Commission or National Medical Product Administration allow personalized phage therapy to take an example of. Such experiences are models for improvement in regulations of phage therapy in China.

In summary, just as how studying phage greatly promoted the advancement in various fields including virology, genetics, molecular biology, and synthetic biology, progress in phage therapy can assist pharmaceutical regulatory authorities to update and optimize related policies, especially regarding personalized therapies that have enormous future potential. Diving into these discussions in this article, we believe that the revolutionary landmarks in phage therapy represent its renaissance in the mainstream of the modern medical field.

## Author contributions

All authors listed have made a substantial, direct, and intellectual contribution to the work and approved it for publication.
